# Sex-dependent mild cardiac dysfunction and neurometabolic abnormalities in a model of Huntington's disease

**DOI:** 10.1242/dmm.052922

**Published:** 2026-09-30

**Authors:** Kenna L. R. Hynes, Thomas M. O'Brien, Jenna Hanrahan, Rachel Neita, T. Nadine Burry, Michael R. Hayden, Matthew P. Parsons, Amber L. Southwell, Lindsay S. Cahill

**Affiliations:** ^1^ Department of Chemistry, Memorial University of Newfoundland, St John's, NL A1C 5S7, Canada; ^2^ Centre for Molecular Medicine and Therapeutics, Vancouver, BC V5Z 4H4, Canada; ^3^ BC Children's Hospital Research Institute, Vancouver, BC V5Z 4H4, Canada; ^4^ Department of Medical Genetics, University of British Columbia, Vancouver, BC V6T 1Z3, Canada; ^5^ Faculty of Medicine, Division of Biomedical Sciences, Memorial University of Newfoundland, St John's, NL A1B 3X7, Canada; ^6^ Burnett School of Biomedical Sciences, University of Central Florida, Orlando, FL 32827, USA; ^7^ Division of Radiology, Memorial University of Newfoundland, St John's, NL A1B 3X7, Canada

**Keywords:** Brain metabolism, Cardiac function, Huntington's disease, Mouse, Sex differences

## Abstract

Huntington's disease (HD) is a neurodegenerative disease with progressive decline in psychiatric, motor and cognitive symptoms. Hallmarks of HD include brain volume loss and abnormal metabolism. However, HD is a multi-system disorder, and the impact on other organ systems is poorly understood. Using high-frequency ultrasound and nuclear magnetic resonance, we studied cardiac function (*n*=9-11 mice/genotype/sex) and brain metabolism (*n*=10 mice/genotype/sex) in a humanized transgenic mouse model of HD (Hu97/18 mice) at 12 months of age. The assessments were conducted in different cohorts and analyzed separately. Compared to controls (Hu18/18 mice), Hu97/18 mice showed a decrease in the left ventricle ejection fraction in both sexes and an increase in the E/A ratio (E wave/A wave diastolic filling) in females, indicative of mild cardiac dysfunction. The relative concentrations of several metabolites in the brain were altered in Hu97/18 mice, compared to those in Hu18/18 mice, and depended on sex and brain region. These findings are consistent with clinical features observed in human HD, supporting use of the Hu97/18 model for investigation of pathophysiology in HD in multiple organ systems. It also highlights the importance of studying both sexes in HD research.

## Teaser

**Editor's choice:** Using biomedical imaging technologies and a humanized transgenic mouse model of Huntington's disease (HD), this work identified peripheral organ dysfunction and neurochemical signatures of HD that were dependent on sex.

## INTRODUCTION

The interconnected relationship between the heart and the brain is critical for understanding the underlying mechanisms that contribute to the progression of neurodegenerative diseases such as Huntington's disease (HD). The most common clinical symptoms of HD are associated with the brain, including progressive psychiatric decline, motor impairment and cognitive deficits (Parsons and Raymond, 2023). Neuroimaging such as structural magnetic resonance imaging (MRI) is often used as a biomarker of HD progression, with volume loss in the striatum occurring early in the disease (Ross et al., 2014). Patients with HD also show evidence of abnormal brain metabolism, including reduction of glucose consumption and decreased N-acetylaspartate (NAA) in the striatum (Kuwert et al., 1989; Sturrock et al., 2010). However, HD is considered a multi-system disorder, with the mutant huntingtin protein found throughout the body (Mielcarek, 2015; Strong et al., 1993). In addition to the brain phenotype, epidemiological studies have reported that patients with HD exhibit abnormalities in cardiac health (e.g. low heart rate variability, arrhythmia), leading to heart failure (Lanska et al., 1988; Stephen et al., 2018; Zielonka et al., 2025). The cause of the cardiac abnormalities (central nervous system dysfunction or abnormal huntingtin protein in the heart) is unknown, and understanding of the bidirectional relationship between the heart and the brain in HD remains incomplete.

Using mouse models, several groups have investigated the effect of HD on brain metabolism or on peripheral organs including the heart. Changes in brain small-molecule metabolites have been demonstrated using nuclear magnetic resonance (NMR) in several mouse models of HD, including R6/2 transgenic HD (Jenkins et al., 2005; Tkac et al., 2012; Zacharoff et al., 2012), *Hdh*Q250 (Jin et al., 2015), YAC128 (Petrella et al., 2018), zQ175 (Heikkinen et al., 2012; Peng et al., 2016) and Ki140CAG (Pépin et al., 2016) mice. Cardiac dysfunction has been reported for male R6/2 and R6/1 mice (Kiriazis et al., 2012; Mihm et al., 2007), BACHD mice (sex not reported) (Schroeder et al., 2011, 2016; Zhu et al., 2020), and female and male R6/2 and *Hdh*Q150 mice (data were not analyzed by sex) (Mielcarek et al., 2014; Wood et al., 2012). To the best of our knowledge, brain metabolism and cardiac function in female HD mice has been understudied.

To better understand the pathophysiology of cardiac function and brain metabolism in HD, a humanized transgenic mouse model of HD was used (Southwell et al., 2013). HD is a monogenetic disease caused by a CAG repeat expansion in the huntingtin (*HTT*) gene. The Hu97/18 mouse model closely mirrors the genetic profile of human patients, particularly the heterozygous HD variant on the human genetic background together with humanization of the unaffected allele, making it highly relevant for translational studies. Hu97/18 mice lack the endogenous mouse *Hdh* (also known as *Htt*) gene and instead express two copies of the full-length human *HTT* gene: one mutant *HTT* with a CAG expansion (97Q) and one wild-type *HTT* (18Q). Hu97/18 mice have a normal lifespan and, by 12 months of age, show significant reductions in striatal and cortical volumes compared to those in Hu18/18 mice (controls that express only human wild-type *HTT*) (Southwell et al., 2018). These changes are similar to the neurodegeneration observed in patients with HD, affecting brain regions that are very metabolically active (Vaishnavi et al., 2010). The goal of this study was to characterize clinical features of HD in this mouse model at 12 months of age, including altered cardiac function and metabolic abnormalities in the brain. This work supports the utility of this mouse model for further investigation of the cardiac and brain phenotypes in HD.

## RESULTS

### Body weights and rotarod performance

The body weight and accelerating rotarod data for the female and male Hu97/18 and Hu18/18 mice are summarized in Table 1. For the body weights at 12 months of age, there were significant main effects of genotype (*P*=0.001) and sex (*P*=0.0009), with the Hu97/18 mice weighing 11% more than the Hu18/18 controls. On the accelerating rotarod test, there was a trend towards a decreased average latency to fall for the Hu97/18 mice compared to Hu18/18 mice (*P*=0.066), and there was no difference between females and males. There was a significant negative relationship between body weight and average latency to fall (*P*=0.04), suggesting that poor rotarod performance is partially related to increased body weight in the Hu97/18 mice (Southwell et al., 2018).

**Table 1.** Body weight and latency to fall in the accelerating rotarod at 12 months of age

Genotype	Sex	Body weight (g)	Latency to fall (s)
Hu18/18	Female	49±4	5.0±3.7
Hu18/18	Male	58±5	7.6±6.4
Hu97/18	Female	58±3	3.3±0.7
Hu97/18	Male	61±3	3.4±0.7

Data are presented as means and 95% c.i. The protocol involved training the mice for 3 days [18 revolutions/min (rpm) for three trials] and testing on the fourth day (increasing the acceleration from 5 to 40 rpm over 5 min, averaging the latency to fall over three tests).

### Abnormal cardiac function in the Hu97/18 mice

The assessments of cardiac function and brain metabolite levels were conducted in independent cohorts; therefore, conclusions are based on parallel comparisons of groups. Cardiac ultrasound parameters were compared with genotype and sex; Table 2 summarizes the significant main effects and interactions (Fig. 1). Heart rate was not significantly different between genotype or sex. As expected, several ultrasound parameters had a main effect of sex and were elevated in males compared to females, including left ventricle area/volume during systole and diastole (*P*<0.006), internal diameter during systole and diastole (*P*<0.01), stroke volume (*P*=0.0008) and cardiac output (*P*=0.001). The E/A ratio (E wave/A wave diastolic filling) was elevated in female mice compared to that in male mice (*P*=0.02). There was a main effect of genotype for the left ventricle systolic area/volume (*P*<0.02), ejection fraction (*P*=0.02) and E/A ratio (*P*=0.01). The ejection fraction was decreased by 10% [female Hu97/18, 60% (95% c.i. 53-67); male Hu97/18, 57% (95% c.i. 51-63); female Hu18/18, 67% (95% c.i. 62-72); male Hu18/18, 64% (95% c.i. 59-69)], and the E/A ratio was increased by 34% [female Hu97/18, 4.1 (95% c.i. 3.1-5.1); male Hu97/18, 2.6 (95% c.i. 1.8-3.4); female Hu18/18, 2.5 (95% c.i. 2.0-3.0); male Hu18/18, 2.5 (95% c.i. 2.1-2.9)], in the Hu97/18 mice compared to that in the Hu18/18 controls. There was a significant genotype-by-sex interaction for the E/A ratio (*P*=0.02). Post hoc analysis showed that the increase in E/A ratio was only in the female Hu97/18 mice (*P*=0.006), with no difference in the E/A ratio between male Hu97/18 and Hu18/18 mice. The corrected left ventricle mass was 16% higher in the Hu97/18 mice than that in the Hu18/18 mice (*P*=0.046), but this difference was not significant after correction for multiple comparisons. There was a significant positive linear relationship between several of the cardiac parameters and body weight, including left ventricle area/volume/mass, stroke volume and cardiac output (*P*<0.02). Although there was no association between body weight and ejection fraction and E/A ratio, obesity was one factor that contributed to the abnormal cardiac function in Hu97/18 mice, explaining 17-19% of the variance in the data (left ventricle systolic area adjusted *R*^2^=0.19, left ventricle systolic volume adjusted *R*^2^=0.17).

**Fig. 1.**
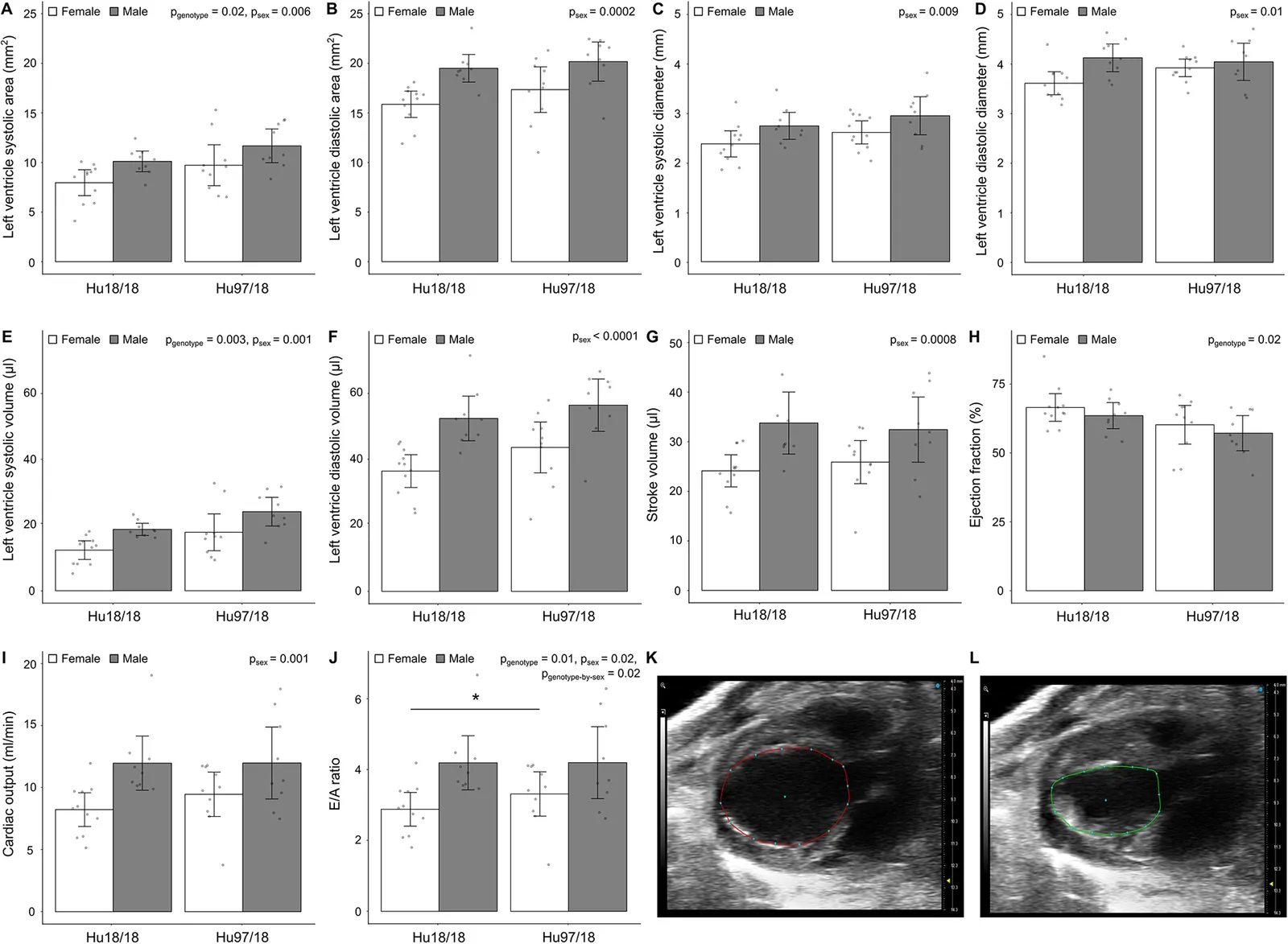
**Cardiac ultrasound.** (A-J) Differences in cardiac parameters (left ventricle systolic area, A; left ventricle diastolic area, B; left ventricle systolic diameter, C; left ventricle diastolic diameter, D; left ventricle systolic volume, E; left ventricle diastolic volume, F; stroke volume, G; ejection fraction, H; cardiac output, I; E/A ratio, J) between female (white) and male (gray) Hu18/18 (control) and Hu97/18 [Huntington's disease (HD)] mice at 12 months of age. Main effects of genotype and sex are noted as ‘p_genotype_’ and ‘p_sex_’, respectively, and genotype-by-sex interaction is noted as ‘p_genotype-by-sex_’. The E wave/A wave (E/A) ratio is a measure of diastolic filling. Data are presented as means and 95% c.i. *P<0.05 by post hoc test. A,B,E-I, *n*=10 female Hu97/18, *n*=9 male Hu97/18, *n*=11 female Hu18/18, *n*=9 male Hu18/18; C,D, *n*=11 female Hu97/18, *n*=9 male Hu97/18, *n*=11 female Hu18/18, *n*=9 male Hu18/18; J, *n*=9 female Hu97/18, *n*=9 male Hu97/18, *n*=8 female Hu18/18, *n*=9 male Hu18/18. (K,L) Representative parasternal long-axis ultrasound views during diastole (K) and systole (L). E/A, E wave/A wave.

**Table 2.** Cardiac function at 12 months of age, with main effects for variables modeled as a two-way interaction between genotype and sex

Ultrasound parameter	Genotype	Sex	Interaction
Heart rate (beats/min)	NS	NS	NS
LV systolic area (mm^2^)	*F*=6.1, *P*=0.02*, *q*=0.09, *ηP^2^*=0.1	*F*=8.6, *P*=0.006*, *q*=0.05, *ηP^2^*=0.2	NS
LV diastolic area (mm^2^)	NS	*F*=16.8, *P*=0.0002**, *q*=0.007, *ηP^2^*=0.3	NS
LV internal diameter in systole (mm)	NS	*F*=7.6, *P*=0.009**, *q*=0.07, *ηP^2^*=0.2	NS
LV internal diameter in diastole (mm)	NS	*F*=7.4, *P*=0.01*, *q*=0.07, *ηP^2^*=0.2	NS
LV systolic volume (μl)	*F*=10.2, *P*=0.003**, *q*=0.03, *ηP^2^*=0.2	*F*=13.0, *P*=0.001**, *q*=0.01, *ηP^2^*=0.3	NS
LV diastolic volume (μl)	NS	*F*=23.0, *P*<0.0001**, *q*=0.006, *ηP^2^*=0.4	NS
Stroke volume (μl)	NS	*F*=13.3, *P*=0.0008**, *q*=0.01, *ηP^2^*=0.3	NS
Ejection fraction (%)	*F*=6.1, *P*=0.02*, *q*=0.09, *ηP^2^*=0.1	NS	NS
Fractional shortening (%)	NS	NS	NS
Cardiac output (ml/min)	NS	*F*=12.2, *P*=0.001*, *q*=0.02, *ηP^2^*=0.3	NS
Corrected LV mass (mg)	NS	NS	NS
LV anterior wall thickness in systole (mm)	NS	NS	NS
LV anterior wall thickness in diastole (mm)	NS	NS	NS
LV posterior wall thickness in systole (mm)	NS	NS	NS
LV posterior wall thickness in diastole (mm)	NS	NS	NS
Mitral isovolumetric relaxation (ms)	NS	NS	NS
Mitral isovolumetric contraction (ms)	NS	NS	NS
Aortic ejection time (ms)	NS	NS	NS
E/A ratio	*F*=7.2, *P*=0.01*, *q*=0.07, *ηP^2^*=0.2	*F*=6.3, *P*=0.02*, *q*=0.09, *ηP^2^*=0.2	*F*=5.9, *P*=0.02*, *q*=0.09, *ηP^2^*=0.2

The uncorrected *P*-values are marked with an asterisk if significant at a false discovery rate of 10% and two asterisks if significant at a false discovery rate of 5%. E/A, E wave/A wave; LV, left ventricle; NS, not significant.

### Metabolite levels varied significantly by brain region

The NMR metabolomics experiments were collected in a separate cohort of mice. Relative metabolite concentrations were compared according to genotype, sex and brain region, and Table 3 summarizes the significant main effects and interactions (Fig. 2). For 17 of the 18 metabolites measured by NMR in the cortex, striatum and cerebellum, there was a significant main effect of brain region. Post hoc tests showed that the relative concentrations of alanine (*P*<0.02), NAA (*P*<0.0001), choline (*P*<0.003), myo-inositol (*P*<0.002), glucose (*P*<0.02) and threonine (*P*<0.0001) were significantly variable across all three brain regions, and how they varied depended on the metabolite. Alanine relative concentration decreased from cortex to striatum to cerebellum, NAA and glucose relative concentrations decreased from cortex to cerebellum to striatum, myo-inositol relative concentration decreased from cerebellum to striatum to cortex, and choline and threonine relative concentrations decreased from striatum to cortex to cerebellum. Additionally, in the cortex, gamma-aminobutyric acid (GABA) relative concentration was significantly elevated compared to that in the striatum (*P*<0.0001) and cerebellum (*P*<0.0001). In the striatum, concentrations of isoleucine/leucine/valine (*P*=0.0005, *P*=0.002) and phosphocholine (*P*<0.0001, *P*<0.0001) were elevated compared to those in the cortex and cerebellum. Aspartate (*P*<0.0001, *P*<0.0001), lactate (*P*=0.001, *P*=0.008), glutamate (*P*=0.0002, *P*=0.02) and glycine (*P*<0.0001, *P*=0.004) concentrations were decreased in the striatum compared to those in the cortex and cerebellum. In the cerebellum, creatine (*P*<0.0001, *P*<0.0001) relative concentration was significantly elevated, and glycerophosphorylcholine (GPC; *P*<0.0001, *P*<0.0001), taurine (*P*<0.0001, *P*<0.0001) and lysine (*P*=0.006, *P*=0.0002) relative concentrations were significantly decreased, compared to those in the cortex and striatum. Significant genotype-by-region interactions were found for isoleucine/leucine/valine (*P*=0.009), lactate (*P*=0.03), GABA (*P*=0.008), creatine (*P*=0.001) and taurine (*P*=0.001). Post hoc tests showed that the relative concentration of GABA was decreased in the Hu97/18 mice compared to that in the Hu18/18 mice in the striatum (*P*=0.002) but not in the cortex or cerebellum, and that creatine (*P*=0.002) and taurine (*P*=0.003) relative concentrations were elevated in the Hu97/18 mice compared to those in Hu18/18 mice in the cerebellum.

**Fig. 2.**
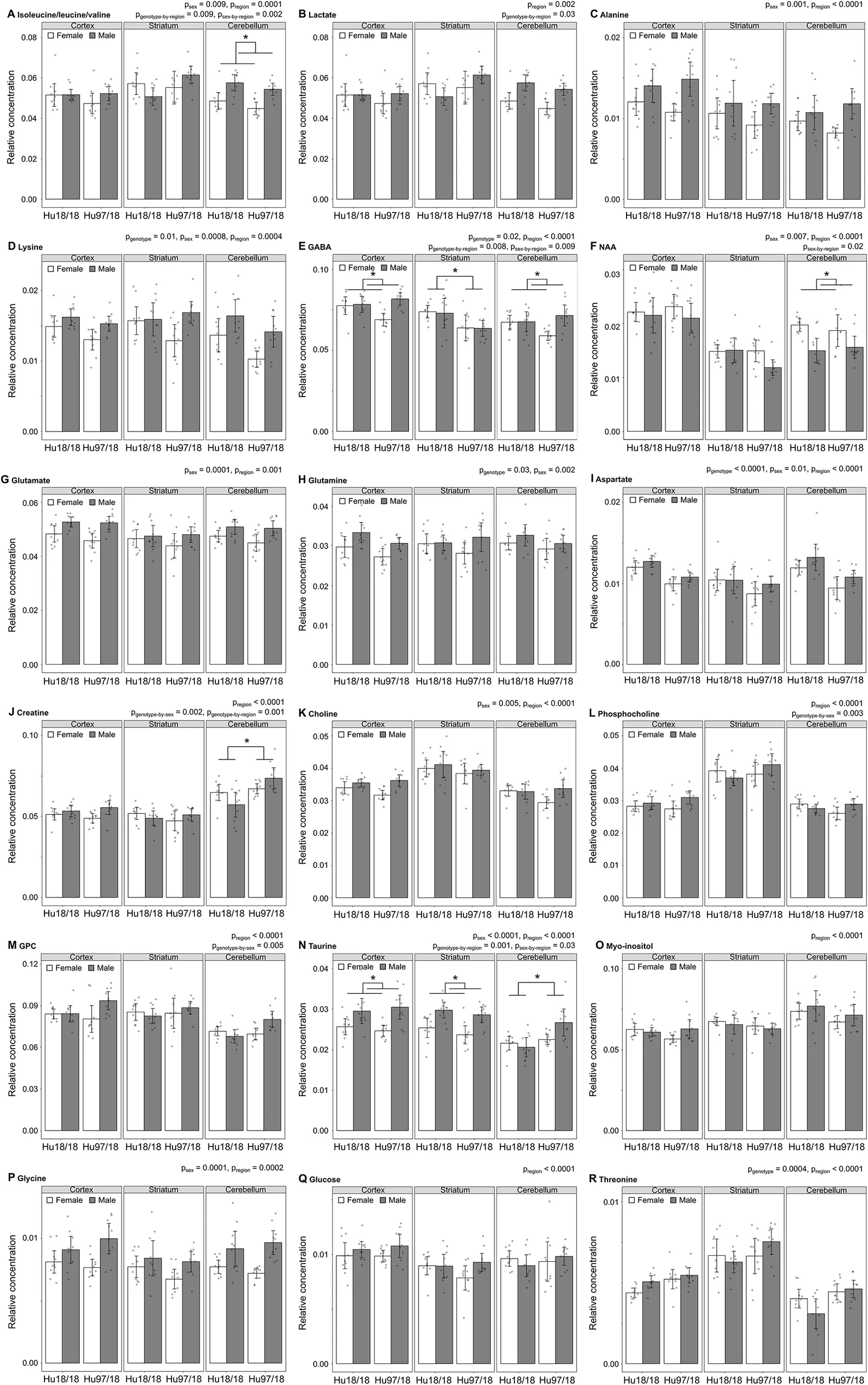
**NMR metabolomics.** (A-R) Differences in relative concentrations of metabolites (isoleucine/leucine/valine, A; lactate, B; alanine, C; lysine, D; GABA, E; NAA, F; glutamate, G; glutamine, H; aspartate, I; creatine, J; choline, K; phosphocholine, L; GPC, M; taurine, N; myo-inositol, O; glycine, P; glucose, Q; threonine, R) between female (white) and male (gray) Hu18/18 (control) and Hu97/18 (HD) mice at 12 months of age. Main effects of genotype, sex and brain region are noted as ‘p_genotype_’, ‘p_sex_’ and ‘p_region_’, respectuvely, and genotype-by-sex, genotype-by-region and sex-by-region interactions are noted as ‘p_genotype-by-sex_’, ‘p_genotype-by-region_’ and ‘p_sex-by-region_’, respectively. *n*=10 mice/genotype/sex. Data are presented as means and 95% c.i. *P<0.05 by post hoc test. GABA, gamma-aminobutyric acid; GPC, glycerophosphorylcholine; NAA, N-acetylaspartate.

**Table 3.** Brain metabolites at 12 months of age, with main effects for variables modeled as a three-way interaction between genotype, sex and brain region

Metabolite	Genotype	Sex	Brain region	Interaction
Isoleucine/leucine/valine	NS	*F*=7.5, *P*=0.009**, *q*=0.03, *R*^2^=0.06	*F*=10.3, *P*=0.0001**, *q*=0.0007, *R*^2^=0.09	Genotype-by-region: *F*=5.0, *P*=0.009**, *q*=0.03, *R*^2^=0.05 Sex-by-region: *F*=6.8, *P*=0.002**, *q*=0.008, *R*^2^=0.07
Lactate	NS	NS	*F*=6.8, *P*=0.002**, *q*=0.008, *R*^2^=0.05	Genotype-by-region: *F*=3.5, *P*=0.03*, *q*=0.1, *R*^2^=0.03
Alanine	NS	*F*=12.6, *P*=0.001**, *q*=0.006, *R*^2^=0.2	*F*=37.9, *P*<0.0001**, *q*=0.0007, *R*^2^=0.2	NS
Lysine	*F*=7.0, *P*=0.01**, *q*=0.04, *R*^2^=0.09	*F*=13.5, *P*=0.0008**, *q*=0.005, *R*^2^=0.2	*F*=8.7, *P*=0.0004**, *q*=0.003, *R*^2^=0.06	NS
GABA	*F*=6.2, *P*=0.02*, *q*=0.06, *R*^2^=0.07	NS	*F*=34.0, *P*<0.0001**, *q*=0.0007, *R*^2^=0.2	Genotype-by-region: *F*=5.1, *P*=0.008**, *q*=0.03, *R*^2^=0.04 Sex-by-region: *F*=5.0, *P*=0.009**, *q*=0.03, *R*^2^=0.04
NAA	NS	*F*=8.1, *P*=0.007**, *q*=0.03, *R*^2^=0.1	*F*=121.7, *P*<0.0001**, *q*=0.0007, *R*^2^=0.5	Sex-by-region: *F*=4.2, *P*=0.02*, *q*=0.06, *R*^2^=0.03
Glutamate	NS	*F*=20.5, *P*=0.0001**, *q*=0.0007, *R*^2^=0.2	*F*=7.5, *P*=0.001**, *q*=0.006, *R*^2^=0.08	NS
Glutamine	*F*=5.3, *P*=0.03*, *q*=0.08, *R*^2^=0.05	*F*=11.7, *P*=0.002**, *q*=0.007, *R*^2^=0.1	NS	NS
Aspartate	*F*=30.0, *P*<0.0001**, *q*=0.0007, *R*^2^=0.2	*F*=7.0, *P*=0.01**, *q*=0.04, *R*^2^=0.07	*F*=13.1, *P*<0.0001**, *q*=0.0007, *R*^2^=0.1	NS
Creatine	NS	NS	*F*=65.8, *P*<0.0001**, *q*=0.0007, *R*^2^=0.5	Genotype-by-sex: *F*=11.9, *P*=0.002**, *q*=0.007, *R*^2^=0.08 Genotype-by-region: *F*=7.5, *P*=0.001**, *q*=0.006, *R*^2^=0.05
Choline	NS	*F*=9.2, *P*=0.005**, *q*=0.02, *R*^2^=0.08	*F*=63.6, *P*<0.0001**, *q*=0.0007, *R*^2^=0.5	NS
Phosphocholine	NS	NS	*F*=130.1, *P*<0.0001**, *q*=0.0007, *R*^2^=0.7	Genotype-by-sex: *F*=10.3, *P*=0.003**, *q*=0.01, *R*^2^=0.08
GPC	NS	NS	*F*=36.9, *P*<0.0001**, *q*=0.0007, *R*^2^=0.3	Genotype-by-sex: *F*=9.0, *P*=0.005**, *q*=0.02, *R*^2^=0.09
Taurine	NS	*F*=30.1, *P*<0.0001**, *q*=0.0007, *R*^2^=0.2	*F*=29.3, *P*<0.0001**, *q*=0.0007, *R*^2^=0.3	Genotype-by-region: *F*=7.3, *P*=0.001**, *q*=0.007, *R*^2^=0.08 Sex-by-region: *F*=3.8, *P*=0.03*, *q*=0.08, *R*^2^=0.04
Myo-inositol	NS	NS	*F*=39.2, *P*<0.0001**, *q*=0.0007, *R*^2^=0.3	NS
Glycine	NS	*F*=21.0, *P*=0.0001**, *q*=0.0007, *R*^2^=0.2	*F*=9.5, *P*=0.0002**, *q*=0.001, *R*^2^=0.07	NS
Glucose	NS	NS	*F*=13.7, *P*<0.0001**, *q*=0.0007, *R*^2^=0.1	NS
Threonine	*F*=15.4, *P*=0.0004**, *q*=0.003, *R*^2^=0.1	NS	*F*=86.8, *P*<0.0001**, *q*=0.0007, *R*^2^=0.6	NS

The uncorrected *P*-values are marked with an asterisk if significant at a false discovery rate of 10% and two asterisks if significant at a false discovery rate of 5%. GABA, gamma-aminobutyric acid; GPC, glycerophosphorylcholine; NAA, N-acetylaspartate; NS, not significant.

### Metabolite levels varied significantly by sex

There was a significant main effect of sex for ten of the 18 metabolites. The relative concentration of NAA was higher in females than that in males (*P*=0.007), and isoleucine/leucine/valine (*P*=0.009), alanine (*P*=0.001), lysine (*P*=0.0008), glutamate (*P*=0.0001), glutamine (*P*=0.002), aspartate (*P*=0.01), choline (*P*=0.005), taurine (*P*<0.0001) and glycine (*P*=0.0001) relative concentrations were higher in males than those in females. There was a trend towards an increase in GABA concentration in males compared to that in females (*P*=0.04). A significant sex-by-region interaction was found for isoleucine/leucine/valine (*P*=0.002), GABA (*P*=0.009), NAA (*P*=0.02) and taurine (*P*=0.03). Post hoc tests showed that the sex differences for NAA (*P*=0.0002) and isoleucine/leucine/valine (*P*<0.0001) were only in the cerebellum, while GABA concentration was elevated in the cortex and cerebellum in males compared to that in females (*P*=0.004, *P*=0.01), and taurine concentration was elevated in the cortex and striatum in males compared to that in females (*P*<0.0001, *P*<0.0001).

### Sex-specific impact on brain metabolism in Hu97/18 mice

There was a significant main effect of genotype for the relative concentrations of lysine (*P*=0.01), GABA (*P*=0.02), glutamine (*P*=0.03), aspartate (*P*<0.0001) and threonine (*P*=0.0004). There was a trend towards genotype differences for the relative concentrations of creatine (*P*=0.04) and myo-inositol (*P*=0.04). The relative concentration of threonine was elevated, whereas aspartate concentration was decreased, in the Hu97/18 mice compared to that in Hu18/18 mice. There was a genotype-by-sex interaction for GABA (*P*=0.04), driven by a decreased relative concentration in the female Hu97/18 mice compared to that in Hu18/18 mice, but this was not significant after correction for multiple comparisons. Significant genotype-by-sex interactions were found for phosphocholine (*P*=0.003), GPC (*P*=0.005) and creatine (*P*=0.002). Post hoc tests showed that the relative concentrations of GPC (*P*=0.002) and creatine (*P*=0.01) were significantly elevated in the male Hu97/18 mice compared to those in Hu18/18 mice, and there were no differences in their concentrations in females. There was a trend towards genotype-by-sex-by-region interaction for GABA (*P*=0.046) and NAA (*P*=0.048). There were no statistically significant associations between body weight and the relative metabolite concentrations, suggesting that the obesity observed in the humanized transgenic mice did not affect the neurometabolic profiles.

### Altered biochemical pathway in the brains of Hu97/18 mice

There was no statistically significant separation of the Hu97/18 and Hu18/18 brain metabolite profiles for the first and second principal components using unsupervised principal component analysis (PCA) (represented 67.9% of the total variation, *P*>0.05). Using supervised partial least squares discriminant analysis (PLS-DA), the overall brain metabolic profile for the Hu97/18 mice was shifted relative to that for the Hu18/18 mice, with a modest separation (*R*^2^=0.26, *Q*^2^=0.14, classification accuracy=0.63) (Fig. 3). The variable importance in projection (VIP; >1.0) lists four metabolites primarily responsible for the differences in brain metabolism observed in the Hu97/18 mice, including GABA, myo-inositol, GPC and creatine. The metabolic pathway analysis revealed perturbations in one biochemical pathway in the Hu97/18 mouse brains (impact factor>0.0): alanine, aspartate and glutamate metabolism (*P*<0.0001, impact score=0.42, match status=3/28).

**Fig. 3.**
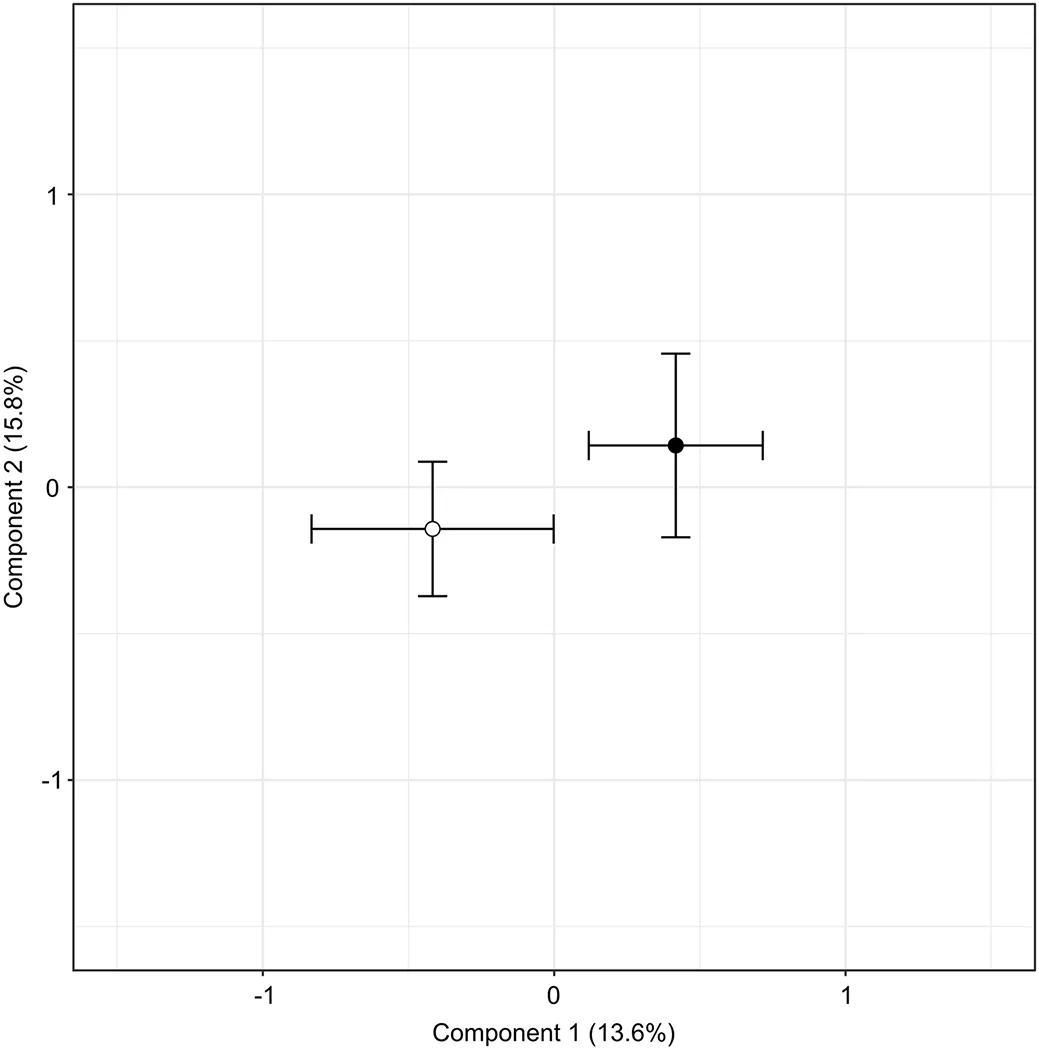
**Averaged partial least squares discriminant analysis two-dimensional scores plot comparing variation in the brain metabolite profile between Hu97/18 mice and Hu18/18 mice.** Black, Hu97/18 mice; white, Hu18/18 mice. *R*^2^=0.26, *Q*^2^=0.14. *n*=10 mice/genotype/sex.

## DISCUSSION

Using high-frequency ultrasound and NMR metabolomics, we identified parallel cardiac and brain phenotypes in a humanized mouse model of HD. Compared to Hu18/18 mice, the Hu97/18 mice showed evidence of systolic and diastolic cardiac dysfunction, with a decreased left ventricle ejection fraction in both females and males and an increased E/A ratio only in the female Hu97/18 mice. The Hu97/18 mice showed significant metabolic abnormalities in the brain, with elevated relative concentrations of threonine in males and females, and GPC and creatine elevated only in males. Lysine, glutamine and aspartate were decreased in all three brain regions (cortex, striatum, cerebellum), and there was an increase in taurine in the cerebellum, and a decrease in GABA localized to the striatum, in Hu97/18 mice compared to that in Hu18/18 mice. In combination, these experimental data help define the physiological and neurometabolic changes in HD and are consistent with clinical features observed in human HD, including a higher rate of cardiovascular events, abnormal intraventricular conduction and abnormal metabolite levels in brain regions impacted in HD (e.g. decrease in striatal GABA) (Patassini et al., 2016; Stephen et al., 2018; Sturrock et al., 2010; Zielonka et al., 2025). These measurements were collected in separate cohorts, and, as a result, the findings demonstrate parallel organ involvement rather than a direct within-animal correlation or mechanistic interaction.

The decreased left ventricle ejection fraction (the percentage of blood pumped in versus out of the heart during a cardiac cycle) in the Hu97/18 mice at 12 months of age is consistent with previous work in R6/2 transgenic mice, in which decreased stroke volume and cardiac output were reported starting at 2 months of age using ultrasound (Mihm et al., 2007) and at 4 months using cardiac MRI (Wood et al., 2012). In contrast, BACHD mice showed no difference in ejection fraction at 8 months of age (Zhu et al., 2020), and had an elevated ejection fraction at 6, 12 and 15 months, compared to that in controls (Schroeder et al., 2016). The trend towards an increased left ventricle mass in Hu97/18 mice is consistent with ultrasound measurements in 9-month-old BACHD mice (Wood et al., 2012). Measurements of heart weight (normalized to tibia length) also increased in R6/2 mice at 1 month of age and in *Hdh*Q150 mice late in life (22 months) (Mielcarek et al., 2014). We observed an increase in the E/A ratio in female Hu97/18 mice, suggesting a restrictive filling pattern resulting from stiffening of the left ventricle. Although we found no difference in E/A ratio between Hu97/18 and Hu18/18 male mice, male R6/2 mice showed a decrease in the E/A ratio from impaired early diastolic relaxation (Mihm et al., 2007). An increased E/A ratio is associated with cardiac failure and has been reported in humans with neurodegenerative diseases including Alzheimer's disease and frontotemporal dementia (Daniele et al., 2020; Yang et al., 2020). It should be noted that the magnitude of the changes observed in the present study (10-34%) suggest only mild cardiac dysfunction compared to the abnormalities reported for the R6/2 mice (e.g. a 50% decrease in cardiac output; Mihm et al., 2007). The average ejection fraction in the Hu97/18 mice (59%) was slightly below the threshold for normal heart function in mice [60-75% (Lindsey et al., 2018), 65-90% (Meng et al., 2024)]. The small effect size of the ejection fraction measure (*ηP^2^*=0.1) might not be physiologically meaningful, and, although there were no differences in the isoflurane anesthesia level or heart rate between genotypes, other factors such as arterial blood pressure were not recorded and may have influenced this measurement.

In addition to the difference in diastolic function between female and male Hu97/18 mice, we observed a sex-specific impact on brain metabolism. In Hu97/18 mice, compared to Hu18/18 mice, males showed alterations in a greater number of metabolites than females, with increases in GPC and creatine throughout the brain only in males. Although studies in patients with HD have reported abnormal concentrations of choline-containing compounds and creatine (Hoang et al., 1998; Jenkins et al., 1993; 1998; Sánchez-Pernaute et al., 1999; Sturrock et al., 2010; van den Bogaard et al., 2014), which indicate membrane turnover, the data were not stratified by sex.

An increase in GPC was reported in R6/2 mice (Tkac et al., 2007, 2012; Zacharoff et al., 2012), and an increase in total creatine was reported in R6/2 (Tsang et al., 2006; Zacharoff et al., 2012), YAC128 (Petrella et al., 2018) and zQ175 (Heikkinen et al., 2012) mice; however, no sex effects were reported. Although differences between females and males have been reported in mouse models of HD (Dorner et al., 2007), sex differences in brain metabolism in HD are understudied, and further work is needed to understand the cause of the differences in GPC and creatine between the sexes. Whereas clinical data suggest that female patients with HD often present with more severe symptoms and faster disease progression than do male patients with the disease (Risby-Jones et al., 2024), numerous studies using HD mouse models indicate that females may be more resilient than males, exhibiting less severe decline in motor function (Cao et al., 2018; Li et al., 2023). The mechanisms underlying these differences often involve sex hormones (e.g. testosterone) and altered metabolic pathways. The Hu97/18 mouse model has some known sex differences in body weight gain and rotarod performance (Southwell et al., 2013). Furthermore, compared to striatum of female mice, striatum of male Hu97/18 mice has been reported to exhibit higher disruption in neuroprotective nitric oxide synthase activity and lower extracellular ascorbate levels, correlating with a more severe phenotype (Dorner et al., 2007; Padovan-Neto et al., 2019). Overall, the more significant impact on brain metabolism in the male Hu97/18 brain may indicate either a compensatory response or failure to maintain normal neurochemical activity, highlighting the importance of considering sex as a critical biological variable in HD research.

Significant regional variation in metabolite concentrations was observed across the cortex, striatum and cerebellum, indicating that each brain region has its own distinct metabolic profile. These results are consistent with the Metabolome Atlas of the Aging Mouse Brain (Ding et al., 2021), which showed, using mass spectrometry, that metabolite profiles differ primarily by brain region rather than by sex. This reflects the different functional and cellular needs of each brain region and demonstrates that these needs are often spatially shared between females and males. Many of the regional differences observed in our data align with those of the healthy old age (59 and 92 weeks) mouse brain (Ding et al., 2021). For example, NAA was highest in the cortex, choline was highest in the basal ganglia/striatum, and myo-inositol was highest in the cerebellum. These regional metabolic differences in the aging brain may be associated with cognitive decline and an increased risk of neurodegenerative diseases. It has been suggested that many of the hallmarks of the aging brain are also indicative of HD pathology (Machiela and Southwell, 2020).

In the Hu97/18 mice, the relative concentrations of several metabolites were found to be altered compared to those in Hu18/18 mice, and several of these changes are similar to those reported in other HD mouse models and in patients with HD (Table 4). The observed decrease in striatal concentrations of GABA, the primary inhibitory neurotransmitter, is consistent with established HD pathology, as reduced GABA levels have been reported in the striatum of R6/2 and zQ175 (Peng et al., 2016; Tsang et al., 2006) mice and in post-mortem human HD brains (Patassini et al., 2016). Decreases in GABA are thought to be associated with the chorea symptoms (involuntary movements) in HD (Crossman, 2000). Similarly, reductions in aspartate have been reported across brain regions in R6/2 mice in comparison to those in controls (Tsang et al., 2006). Although the concentration of lysine, a ketogenic amino acid, is not typically associated with HD, the decrease we observed here does mirror amino acid depletion in human HD brains, likely reflecting catabolic stress and a need for tricarboxylic acid and urea cycle substrates (Patassini et al., 2016). These changes, along with the observed perturbations to the biochemical pathway related to alanine, aspartate and glutamate metabolism, highlights dysregulation of amino acid metabolism commonly affected across the human condition and mouse models of HD (Pradhan et al., 2022). Some of the observed metabolite changes highlight differences between mouse models and human HD. Elevated threonine in our mice contrasts with human post-mortem analyses, in which threonine is often decreased (Patassini et al., 2016). In humans, a meta-analysis of proton magnetic resonance spectroscopy (^1^H MRS) studies reported reduced myo-inositol in patients with premanifest HD, suggesting that impaired synthesis of myelin lipids is an early event in the development of disease pathology (Jing et al., 2024). Myo-inositol levels have also been shown to correlate with HTT CAG repeat length in the human putamen, linking this metabolite to disease severity (Patassini et al., 2016). Although some late-stage mouse models also show elevated myo-inositol (Datson et al., 2017; Jing et al., 2024; Zarate et al., 2023), reflective of neuroinflammation, the trend towards a decrease in myo-inositol in our Hu97/18 mice suggests a metabolic profile that more closely resembles early HD. Patients with HD and HD mouse models typically showed increased concentrations of glutamine (Jin et al., 2015; McGarry et al., 2024; Tsang et al., 2006; Zacharoff et al., 2012); however, the relative concentration of glutamine was decreased in our Hu97/18 mice. Although this finding was unexpected, it supports existing literature that the glutamatergic system is compromised in HD. Overall, these comparisons show that, although metabolic dysregulation is a consistent indication of HD, species-specific and disease-stage-dependent differences must be carefully considered when interpreting findings across mouse models and human HD. The Hu97/18 model captures many aspects of human HD metabolomics, highlighting its potential for studying brain health and disease.

**Table 4.** Phenotypes observed in this study and how they compare to existing literature in humans and mice

Reference(s)	Subjects	Phenotype summary
**Cardiac function**		
This study	Hu97/18 mice (females and males)	↓ LV ejection fraction (12 months)
Mihm et al., 2007; Wood et al., 2012	R6/2 mice (males)	↓ Stroke volume (4 months) ↓ Cardiac output (2 months)
Zhu et al., 2020	BACHD mice (females and males)^‡^	No difference in ejection fraction (8 months)
Schroeder et al., 2016	BACHD mice*	↑ Ejection fraction (6, 12, 15 months)
This study	Hu97/18 mice (females and males)	↑ E/A ratio (females only, 12 months)
Mihm et al., 2007	R6/2 mice (males)	↓ E/A ratio (2-3 months)
Daniele et al., 2020; Yang et al., 2020	Human patients (neurodegenerative diseases)	↑ E/A ratio
This study	Hu97/18 mice (females and males)	Trend towards ↑ LV mass (12 months)
Schroeder et al., 2016	BACHD mice*	↑ LV mass (9 months)
Mielcarek et al., 2014	R6/2 and *Hdh*Q150 mice (females and males)^‡^	↑ Heart weight (normalized to tibia length) (R6/2, 1 month) (*Hdh*Q150, 22 months)
**Brain metabolism**		
This study	Hu97/18 mice (females and males)	↓ Lysine (12 months; cortex, striatum, cerebellum)
Patassini et al., 2016	Human HD brains	↓ Lysine (cortex, hippocampus, striatum)
This study	Hu97/18 mice (females and males)	↓ GABA (12 months; localized to striatum)
Peng et al., 2016	zQ175 mice (females and males)^‡^	↓ GABA (12 months; striatum)
Tsang et al., 2006	R6/2 mice*	↓ GABA (starting at 2-3 months, striatum and cerebellum)
Patassini et al., 2016	Human HD brains	↓ GABA (cortex, striatum, cerebellum)
This study	Hu97/18 mice (females and males)	Trend towards ↓ NAA (12 months; male, localized to striatum)
Pépin et al., 2016	R6/1 mice (females and males)^‡^	↓ NAA (12 months; striatum)
Jenkins et al., 2005; Tkac et al., 2007; Tsang et al., 2006; Zacharoff et al., 2012	R6/2 mice*	↓ NAA (starting at 1-3 months; cortex, striatum, cerebellum)
Heikkinen et al., 2012	zQ175 mice (females)	↓ NAA (starting at 4 months; striatum)
Sturrock et al., 2010	Human patients with HD	↓ NAA (striatum)
Sánchez-Pernaute et al., 1999	Human patients with HD	↓ NAA (striatum)
This study	Hu97/18 mice (females and males)	↓ Glutamine (12 months; cortex, striatum, cerebellum)
Tsang et al., 2006; Zacharoff et al., 2012	R6/2 mice*	↑ Glutamine (starting at 2 months; cortex, striatum, cerebellum)
Jin et al., 2015	HdhQ250 mice (males)	↑ Glutamine (12 months; striatum)
McGarry et al., 2024	Human patients with HD	↑ Glutamine
This study	Hu97/18 mice (females and males)	↓ Aspartate (12 months; cortex, striatum, cerebellum)
Tsang et al., 2006	R6/2 mice*	↓ Aspartate (3 months; whole brain)
This study	Hu97/18 mice (females and males)	↑ Creatine (males only)
Tsang et al., 2006; Zacharoff et al., 2012	R6/2 mice*	↑ Total creatine (starting at 2-3 months; cortex, striatum, cerebellum)
Petrella et al., 2018	YAC128 mice (females and males)	↑ Total creatine (9 months; striatum)
Heikkinen et al., 2012	zQ175 mice (females)	↑ Total creatine (12 months; striatum)
Sánchez-Pernaute et al., 1999; Sturrock et al., 2010; van den Bogaard et al., 2014	Human patients with HD	↓ Total creatine (striatum)
Hoang et al., 1998	Human patients with HD	↑ Total creatine (striatum)
This study	Hu97/18 mice (females and males)	↑ GPC (males only)
Tkac et al., 2007; Tkac et al., 2012; Zacharoff et al., 2012	R6/2 mice*	↑ GPC (starting at 2-3 months; cortex, striatum, cerebellum)
Hoang et al., 1998	Human patients with HD	↑ GPC (striatum)
This study	Hu97/18 mice (females and males)	↑ Taurine (localized to cerebellum)
Jenkins et al., 2005; Tkac et al., 2007; Tsang et al., 2006; Zacharoff et al., 2012	R6/2 mice*	↑ Taurine (starting at 2-3 months; cortex, striatum, cerebellum)
Jin et al., 2015	*Hdh*Q250 mice (males)	↑ Taurine (12 months; striatum)
Peng et al., 2016	zQ175 mice (females and males)^‡^	↑ Taurine (12 months; striatum)
This study	Hu97/18 mice (females and males)	Trend towards ↓ myo-inositol (12 months; cortex, striatum, cerebellum)
Datson et al., 2017	R6/2 mice (females and males)	↑ Myo-inositol (3 months; striatum)
Zarate et al., 2023	zQ175 mice (females and males)	↑ Myo-inositol (6 months; striatum)
Patassini et al., 2016	Human HD brains	↓ Myo-inositol (cortex, cerebellum)
van den Bogaard et al., 2014	Human patients with HD	↓ Myo-inositol (striatum)
Jing et al., 2024	Meta-analysis of patients with premanifest HD	↓ Myo-inositol
This study	Hu97/18 mice (females and males)	↑ Threonine (12 months; cortex, striatum, cerebellum)
Patassini et al., 2016	Human HD brains	↓ Threonine (cortex, hippocampus, cerebellum)

*Sex not reported. ^‡^Data not analyzed by sex. HD, Huntington's disease.

The present study has several limitations. The cardiac ultrasound and the brain metabolomics were collected in separate cohorts of mice, preventing the determination of a relationship between heart and brain function in the same mouse. Moreover, both datasets were collected at only one time point, late in the disease progression. A longitudinal study design would allow for monitoring of the temporal dynamics within each mouse and provide important information about causality. Conducting experimental procedures that have known circadian regulation (e.g. cardiac function, brain metabolism) throughout the light phase, rather than at the same time each day, may contribute to variability in the data. Additionally, CAG repeat length was not confirmed in the experimental animals, and potential repeat instability could also contribute to variability. Another limitation is that the use of NMR for metabolomics only allows for the detection of 18 different metabolites. Mass spectrometry provides metabolic information that is complementary to NMR, and several studies have used this technique to detect a larger number of abnormal metabolites and lipids in HD mouse models (Hashimoto et al., 2021; Pfalzer et al., 2019; Vodicka et al., 2015). Both the Hu97/18 and Hu18/18 mice are obese (Southwell et al., 2018), limiting the assessment of motor function and potentially impacting metabolism compared to that in wild-type mice. Obesity is not a phenotype in human HD, with unintended weight loss a hallmark of the disease (Aziz and Roos, 2013). Finally, the brain regions of interest in the present study only included the striatum, cortex and cerebellum. Future studies should include additional regions such as the hippocampus, hypothalamus and medulla, which are known to undergo structural changes in HD mouse models (Hanrahan et al., 2024) and human HD (Petersén and Gabery, 2012; Wilkes et al., 2023), and are involved in contractility and autonomic function in the heart (Critchley et al., 2018).

In summary, this study provided confirmatory evidence for the use of the Hu97/18 mouse model to further investigate the pathophysiology and the cardiac and brain phenotypes in HD. We speculate that the observed metabolic abnormalities in the brain may place the heart under greater stress to maintain cardiac function, resulting in the observed systolic and diastolic dysfunction. Conversely, abnormal heart function may decrease cerebral blood flow and cause cerebral hypoxia, resulting in dysregulation of metabolites critical for proper brain function. In support of this, subclinical cardiac dysfunction (e.g. decreased left ventricle ejection fraction) has been associated with decreased cerebral blood flow and molecular evidence of neurodegeneration (Kresge et al., 2020). The present study identified several promising metabolic signatures of HD (lysine, GABA, glutamine, aspartate, threonine), and future work should investigate the time course and natural history of these metabolites and associated proteins in cerebrospinal fluid and serum (Silajdžić and Björkqvist, 2018). This will be critical for determining whether the observed changes represent primary pathogenic mechanisms contributing to HD or secondary consequences of disease progression. In addition to the importance of abnormal brain chemistry in HD, considerations of cardiac mortality should be included when developing treatment options for patients and determining treatment effectiveness. Future studies should integrate cardiac and brain measurements in the same animals to explore the interconnection and to determine whether the cardiac dysfunction or brain metabolic abnormalities emerge first.

## MATERIALS AND METHODS

### Animal model

Male and female Hu97/18 and Hu18/18 mice were provided by Michael Hayden's laboratory (University of British Columbia, Vancouver, BC, Canada) (Southwell et al., 2013). The mice were bred in house (Hu97/18 crossed with Hu18/18) and co-housed in standard cages with *ad libitum* access to food and water. The housing room was on a 12 h light-dark cycle (lights on at 07:00), and all experimental procedures were conducted during the light phase (09:00-17:00). The estrous cycle stage was not monitored in the female mice (Dalla et al., 2024). All animal experiments performed were approved by the Institutional Animal Care Committee at Memorial University of Newfoundland (Animal Use Protocol 23-02-LC) and conducted in accordance with guidelines established by the Canadian Council of Animal Care. At weaning, tail samples were collected for genotyping.

### Genotyping

DNA was extracted by adding 250 μl of 50 mM NaOH to the tail sample in a 1.5 ml tube and incubated at 98°C for 30 min on a dry heat block. Samples were then spun down at 15,000 ***g*** for 5 min at 20°C. 20 μl supernatant was diluted into 80 μl of 1× Tris-EDTA. 2 μl diluted DNA samples were spot checked with a Nanodrop spectrophotometer (Thermo Fisher Scientific) to ensure that DNA was present. Primers used for PCR amplification were as follows: forward, 5′-ATTGCCCCGGTGCTGAGC-3′; reverse, 5′-GCGGGCCCAAACTCACGGTC-3′. PCR amplification was done with Platinum SuperFi II TAQ (Invitrogen, 12361010), and reagent volumes and thermal cycler conditions used were as stated by the Invitrogen Platinum Super Fi II TAQ product insert. PCR products were run on a 1.5% agarose gel with 10% SYBRsafe (Thermo Fisher Scientific, s33102). The agarose gels were imaged with a Bio-Rad ChemiDoc XRS+ Gel Imaging System.

### Experimental design

The Hu97/18 mice showed overt symptoms of disease [e.g. depressive-like behavior, increased anxiety, decreased motor performance, cognitive deficits (Southwell et al., 2013, 2017, 2018)] starting at 3-6 months of age, and these became more pronounced by 9 months (equivalent to middle-aged humans). To characterize the mice during the manifest stage of disease, the mice were evaluated at 12 months of age. Motor performance was evaluated using an accelerating rotarod [17 Hu97/18 (nine females, eight males) and 14 Hu18/18 (five females, nine males)]; cardiac function was assessed using high-frequency ultrasound [20 Hu97/18 (11 females, nine males) and 20 Hu18/18 (11 females, nine males)]; and, in a separate cohort of mice, brain metabolism was assessed using fixed tissue and solid-state magic angle spinning (MAS) NMR [20 Hu97/18 (ten females, ten males) and 20 Hu18/18 (ten females, ten males)]. Animals were randomized to experimental assays. All experimental procedures including rotarod testing, tissue dissection, ultrasound acquisition and analysis, NMR processing and statistical analyses were performed unaware of genotype and sex. Litter and cage variables were not included in the statistical analyses. No animals or data points were excluded from the NMR metabolomics data. For cardiac ultrasound, one parasternal long-axis (PSLAX) image and five apical four-chamber images were excluded owing to poor image orientation or quality (see ‘Ultrasound assessment of cardiac function’ section).

### Rotarod testing

To assess motor function, an accelerating rotarod (Harvard Apparatus) was used in the week before ultrasound imaging. The mice were trained on the rotarod for three consecutive days at a fixed speed of 18 revolutions/min (rpm) for three trials (2 min/trial). If the mice fell from the rod, they were immediately placed back on for the remainder of the training session. The fourth consecutive day involved placing the mice on the rotarod and increasing the acceleration from 5 to 40 rpm over 5 min. The latency to the first fall was recorded using the timer on the rotarod device. Mice received three tests, and the latency to fall was averaged.

### Ultrasound assessment of cardiac function

Mice were anesthetized using isoflurane (4% for induction and 2% for maintenance) in 100% oxygen and placed supine on a heated ultrasound platform (temperature maintained between 35 and 37°C). Internal temperature, heart rate and respiration were monitored throughout the imaging session to ensure physiological wellbeing. Although the heart rate remained constant during the imaging session, it was lower than that in awake mice [∼350 beats/min versus 400-650 beats/min (Lindsey et al., 2018)]. Hair was removed from the chest using Nair to improve contact with the transducer. Cardiac ultrasound measurements were taken at three different angles – PSLAX, short-axis (SAX) and the apical four-chamber – using a high-frequency ultrasound system (F2, VisualSonics, Toronto, ON, Canada), a 29 MHz transducer and established guidelines (Lindsey et al., 2018; Zacchigna et al., 2021). Briefly, two-dimensional B-mode images of the PSLAX of the heart were collected to allow assessment of left ventricular systolic and diastolic function, specifically measurements of chamber area/volume during systole and diastole, stroke volume, ejection fraction and cardiac output. M-mode images of the endocardium and epicardium of the left ventricle from the SAX view were used to calculate internal diameters during systole and diastole, left ventricle anterior and posterior wall thickness, fractional shortening and left ventricle mass. Using the four-chamber view, pulsed-wave Doppler images were collected at the apex of the heart to assess mitral valve blood flow and diastolic heart function, specifically measurements of trans-mitral blood flow in early diastole (E wave), trans-mitral blood flow from atrial contraction at the end of diastole (A wave), isovolumetric contraction time, aortic ejection time and isovolumetric relaxation time. For one Hu97/18 mouse, the PSLAX image was not in the correct orientation and was excluded. For five of the mice (two Hu97/18 and three Hu18/18), the A and E waves could not be accurately measured owing to low-quality images in the four-chamber view, or the E and A waves were not sufficiently separated in the Doppler waveforms.

### Solid-state MAS NMR metabolomics

The mice were euthanized by cervical dislocation using isoflurane, and the brains were removed and immediately flash frozen in liquid nitrogen and stored at −80°C. The frozen brains were dissected, using landmarks from the Allen Mouse Brain Atlas, into the cerebral cortex, striatum and cerebellum with a cold scalpel and a brain matrix kept on dry ice throughout the dissection. These brain regions were chosen because they are known to be impacted to varying degrees in mouse models of HD and patients with HD. The NMR sample preparation and experiments were conducted as described previously (Neita et al., 2025). Measurements were carried out using a Bruker NEO 600 MHz spectrometer (^1^H Larmor frequency of 600.29 MHz) and a Bruker 4 mm broadband dual-channel high-resolution MAS NMR probe (iProbe). No chemical extraction was performed. Intact, unprocessed tissue samples were transferred directly into disposable Kel-F inserts (Cortecnet; 25 μl) in 4 mm zirconium rotors, which were spun at a rate of 4000 Hz. To suppress the water resonance and signals from lipids that dominate the ^1^H spectra of biological tissues, a Carr–Purcell–Meiboom–Gill experiment with water presaturation was used for the acquisition. The ^1^H one-dimensional experiments were executed with a 90° pulse length of 5 μs, a recycle delay of 2 s and an echo time of 25 μs (total echo time of 64 ms), accumulating 32 scans per experiment. The bearing gas temperature was maintained at 37°C to closely mimic physiological conditions and further reduce line widths. To maintain quality assurance, sample acquisition order was randomized across genotypes, biological sexes and brain regions. Prior to running each sample, system calibrations including pulse width calibrations, magnetic field shimming and automated probe tuning were performed. Sample stability and bearing gas temperature were monitored throughout every acquisition to maintain spectral quality across all samples.

### Ultrasound data processing

Cardiac images were analyzed offline using the VisualSonics VevoLab software (version 5.11.1). All measurements were averaged over three cardiac cycles. The endocardium and epicardium were manually traced during diastole and systole to get measurements of area, volume, diameter and wall thickness. These measurements were used to derive the following:

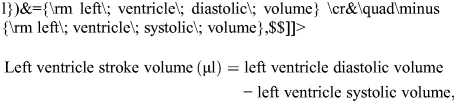


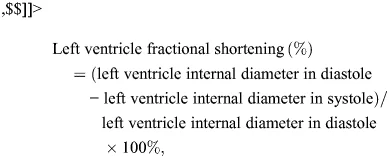


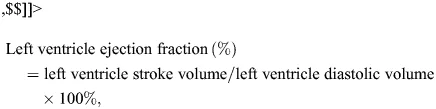


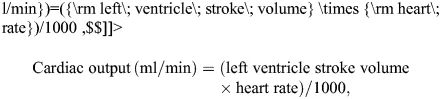


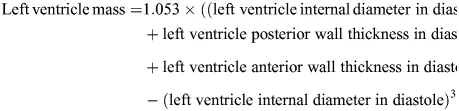
and

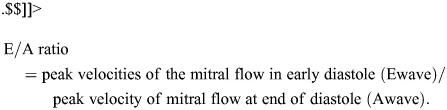


### NMR data processing

^1^H NMR spectra were processed using MestReNova (version 15.0.1). After selecting all spectra, baseline correction was applied using the Whittaker Smoother function, and manual phase correction was used if needed. Chemical shift assignments were made using literature values (Govindaraju et al., 2000; Neita et al., 2025; Tugnoli et al., 2004) and two-dimensional ^1^H-^1^H correlation spectroscopy. No exogenous chemical shift standard or internal reference was added to the tissue. Instead, all spectra were referenced to the endogenous NAA peak at 2.03 ppm to ensure consistent chemical shift alignment. Automated peak picking was performed, and line fitting was used selectively to refine peak shapes when needed, with care taken to avoid over adjustment. Metabolite concentrations are reported as relative concentrations expressed as normalized spectral integrals. The region corresponding to the residual water signal was excluded. For quantification, metabolite peaks of interest were manually integrated using spectral deconvolution. Finally, individual peak integrals were normalized to the total spectral area (0-6 ppm, excluding the water resonance) to account for differences in tissue mass and water content across samples. Additionally, the overlapping signals of isoleucine, leucine and valine were integrated as a single composite peak, as overlapping signals could not be resolved cleanly into individual metabolites.

### Statistical analysis

R statistical software was used for statistical analysis. Data are presented as means and 95% c.i. For body weights, rotarod behavioral testing and cardiac ultrasound parameters, two-way ANOVA was used to evaluate the effect of genotype (Hu97/18, Hu18/18) and biological sex (female, male). If the ANOVA was significant, post hoc tests were performed. For the NMR metabolomics data, a linear mixed effects model was used, to account for the repeated measures of brain regions from the same mouse, with main effects of genotype (Hu97/18, Hu18/18), sex (female, male) and brain region (cortex, striatum, cerebellum), and including interactions terms and mouse ID as the random effect. If the linear mixed effects model was significant for brain region or interactions, additional post hoc tests were performed by relevelling the model with different experimental groups as the reference group and using the lmerTest package and paired two-tailed *t*-tests. Partial eta squared (*ηP^2^*) was used to quantify the size of the main effects as well as the interactions. For the linear mixed effect models, effect sizes (*R*^2^) were calculated using the Snijders and Bosker approach (Snijders and Bosker, 2011). Multiple comparisons were corrected using a false discovery rate of 10% and the p.adjust() function in R (Benjamini and Hochberg, 1995). A false discovery rate of 10% is considered appropriate for imaging and metabolomics studies when the goal is novel hypothesis generation, and 5% is used for more rigorous multiple comparison control (Alka et al., 2022; Schwartzman et al., 2009; Shanthamoorthy, 2022). Differences with an uncorrected *P*<0.05 that were not significant after correction for multiple comparisons are interpreted as showing a trend towards significance. In addition to investigating the differences in individual metabolites, the entire metabolic profiles were compared using unsupervised PCA, supervised PLS-DA and metabolic pathway enrichment analysis in MetaboAnalyst (version 6.0) (Pang et al., 2024). Fivefold cross-validation was used to select the optimal number of classification components used in the PLS-DA model, and, to validate the outcomes, permutation testing was used (using 1000 data resampling steps). VIP scores >1.0 were identified as the metabolites most important to explain the separation in PLS-DA. The pathway analysis was conducted using the list of metabolites that were significantly different between Hu97/18 and Hu18/18 mice, the Kyoto Encyclopedia of Genes and Genomes (KEGG) ID and the *Mus musculus* library. The impact score and match status (hit/total) are reported to represent the proportion of the metabolic pathway that is altered. Only pathways with an impact score >0.0 are considered of biological importance.

## Data and resource availability

All relevant data and details of resources can be found within the article and its supplementary information.
